# Exploring the Predictors of Physical Activity in Older Adults in South Korea Using the Health Belief Model

**DOI:** 10.3390/bs16040547

**Published:** 2026-04-06

**Authors:** Hyungsook Kim, Ye Hoon Lee, Yonghyun Park

**Affiliations:** 1 Department of Data Science, Hanyang University, Seoul 04763, Republic of Korea; khsook12@hanyang.ac.kr; 2 HY-Digital Healthcare Center, Hanyang University, Seoul 04763, Republic of Korea; 3 Division of Global Sport Industry, Hankuk University of Foreign Studies, Yongin 17035, Republic of Korea

**Keywords:** elderly, exercise, health behavior change, perceived barrier, self-efficacy

## Abstract

This study aimed to examine the associations of Health Belief Model (HBM) constructs with physical activity (PA) participation intention and self-reported PA participation among older adults in South Korea. Specifically, we examined whether perceived susceptibility, perceived severity, perceived benefits, perceived barriers, and self-efficacy were associated with PA participation intention and PA participation, and whether intention accounted for indirect associations between HBM constructs and PA within the hypothesized model. A total of 408 older adults (*M*age = 68.84, *SD* = 4.11) participated in the online survey. This study employed Structural Equation Modeling to examine the interrelationships among the proposed variables. The findings indicated a significant negative association between perceived barriers and PA participation intention and a significant positive association between self-efficacy and PA participation intention. Furthermore, intention was positively associated with PA and accounted for indirect associations linking perceived barriers and self-efficacy with PA. Overall, these findings suggest that perceived barriers and self-efficacy are salient belief domains linked to PA intention and behavior. Practical implications include further interventions to reduce perceived barriers and enhance self-efficacy to promote sustained PA engagement among older adults.

## 1. Introduction

The global population is aging rapidly, with the number of individuals aged 65 years and older projected to more than double from 727 million in 2020 to 1.5 billion by 2050, representing 16% of the world’s population (United Nations, Department of Economic and Social Affairs, Population Division, 2019). This demographic shift is particularly pronounced in Asia and Europe, where older adults constitute over 20% of the population, creating significant implications for healthcare systems and economic stability (World Health Organization, 2015). In South Korea, medical expenses for individuals aged over 65 years amounted to 48.9 trillion KRW, representing 44.1% of total national healthcare expenditures (Health Insurance Review and Assessment Service, 2020; Y. Kim, 2020). Additionally, the prevalence of chronic diseases reached 18.8 million in 2019, including 12 major conditions such as hypertension, arthritis, mental and behavioral disorders, neurological diseases, diabetes, and liver diseases (Shin, 2019). Thus, maintaining older adults’ physical function, quality of life, and independence is imperative for both public health and economic stability (Piercy et al., 2018).

Physical activity (PA) is essential to healthy aging as it has the potential not only to maintain quality of life and fitness, but also to sustain the capacity to engage in everyday activities (Niyazi et al., 2024; Piercy et al., 2018; Valenzuela et al., 2023; World Health Organization, 2020). Over the years, several studies have confirmed the benefits of physical activity for the aging process. Promoting physical activity for older adults can help improve and support cardiorespiratory fitness, as well as muscle strength, flexibility, balance, and mobility, thereby contributing to independence and functional capacity (Piercy et al., 2018; World Health Organization, 2020). Consequently, higher levels of physical activity are strongly associated with a reduced risk of both falls and fractures (Edholm et al., 2019; Valenzuela et al., 2023). In addition, PA is linked to the prevention and management of cardiovascular and metabolic conditions and is associated with better mental health outcomes, including lower depressive symptoms and cognitive decline risk (Cunningham et al., 2020; Iso-Markku et al., 2024; Noetel et al., 2024; Yao et al., 2021). However, only a small percentage of the general population currently meets these recommendations, and older adults are the least likely to meet the criteria (Bull et al., 2020), which can increase the risk of chronic and mental illnesses, disability, reduced functional capacity, low quality of life, and high medical costs, creating a major public health problem (Burini et al., 2020; Carlson et al., 2018; Patterson et al., 2018; Silva et al., 2020). This low adherence to recommended PA levels underscores the need to identify factors that are associated with PA participation among older adults and to better understand what motivates long-term participation in healthy aging-related activities.

The Health Belief Model (HBM) is a conceptual framework that explains and predicts health behavior in the population (Rosenstock, 1990). The five major components of HBM (i.e., perceived susceptibility, perceived severity, perceived benefits, perceived barriers, and self-efficacy) have been found to associate with PA participation in prior research (Çiftci & Kadıoğlu, 2023; Sas-Nowosielski et al., 2013; Üstün et al., 2020). Although HBM was originally developed to explain preventive actions aimed at disease avoidance, it is also appropriate for understanding PA in older adulthood because PA is commonly framed as a health-maintenance and disease-prevention behavior in later life (e.g., reducing cardiometabolic risk, preventing functional decline, and maintaining independence) (Piercy et al., 2018; World Health Organization, 2020). HBM provides a parsimonious structure to examine modifiable health beliefs (perceived threat, benefits, barriers, and self-efficacy) that can be directly targeted through counseling, health education, and program design, making it useful for identifying actionable leverage points for PA promotion among older adults. Importantly, HBM is conceptually compatible with alternative frameworks commonly used in PA research. For example, the self-efficacy construct aligns closely with Social Cognitive Theory (Bandura, 1997), and the belief to intention pathway is consistent with intention-based motivational models such as the Theory of Planned Behavior (TPB; Ajzen, 1991). We selected HBM because it offers a theory-driven yet practical framework to test a belief-based pathway model (beliefs–intention–PA) in an understudied population (older adults in South Korea). Despite its broad applicability, gaps remain in HBM research concerning older adults and PA, particularly in non-Western contexts. Most existing studies focus on younger or mixed-age populations, leaving limited knowledge about the unique barriers, motivations, and needs of older adults (S.-E. Kim et al., 2024; Qiao et al., 2021). This research gap is critical, as the components of an individual’s health beliefs can vary significantly based on factors such as age, sex, income, and social status, meaning they do not function uniformly (Alyafei & Easton-Carr, 2024). Additionally, constructs like perceived susceptibility and severity have shown mixed associations with PA-related outcomes in older adults and remain underexplored in South Korea, where aging represents a major public health issue (Shin, 2019).

Accordingly, this study tested an HBM-based pathway model using structural equation modeling (SEM) in older adults in South Korea. Specifically, we examined whether perceived susceptibility, perceived severity, perceived benefits, perceived barriers, and self-efficacy were associated with PA participation intention, and whether intention in turn was associated with actual PA participation. We also tested whether participation intention statistically mediated the associations between the HBM constructs and physical activity behavior in this cross-sectional dataset.

## 2. Literature Review

### 2.1. HBM

HBM was originally developed in the 1950s by social psychologists to explain why individuals did not participate in programs for the early detection of asymptomatic chronic disease (Rosenstock, 1974). Rather than focusing exclusively on individual beliefs, the HBM attempts to capture and predict the likelihood that a person or population will engage in a specific health behavior based on both individual and sociocultural beliefs, factors, and influences (Champion & Skinner, 2008; Green et al., 2020). It has frequently been applied by health researchers when developing health education programs or planning health campaigns. According to HBM, individuals who perceive themselves as susceptible to disease, believe the disease is severe, recognize the benefits of preventive actions, and encounter few obstacles to those actions are more likely to participate in behaviors aimed at disease prevention (Janz & Becker, 1984). Health behavior refers to actions taken by individuals who consider themselves healthy to detect and prevent diseases in the absence of symptoms, and HBM identifies the factors influencing such behaviors (Rosenstock, 1974). Essentially, HBM was created to explain why people manage their health under specific circumstances and for particular reasons (Champion & Skinner, 2008). It posits that human behavior is shaped not by past experiences but by subjective perceptions, with the model encompassing the following components.

HBM comprises four key components: perceived susceptibility, perceived severity, perceived benefit, and perceived barrier (Janz & Becker, 1984). First, perceived susceptibility refers to an individual’s subjective perception of the likelihood of developing a particular disease. This perception is influenced by environmental, demographic, and psychological factors, which shape how threatened an individual feels by disease. The higher the perceived susceptibility, the higher the likelihood that an individual will be involved in health-promoting behaviors. Second, perceived severity refers to an individual’s perception of the seriousness of a disease’s outcomes. These outcomes include perceived mortality risks, disability, pain, and the disease’s effects on family life, work, and social limits. Greater perceived severity increases the likelihood of adopting preventive or coping behaviors. Third, perceived benefits refer to the degree to which an individual believes that participation in health-related behaviors reduces the negative consequences of a particular disease. It also involves the perceived effectiveness of the behavior in maintaining health, preventing disease, or enabling early detection. Greater perceived benefits enhances participation in health-promoting behavior. Fourth, perceived barriers refer to obstacles individuals perceive when performing health behaviors. Barriers may include economic costs, physical or psychological discomfort, and social inconveniences, such as side effects, pain, or other preventive activity-related discomforts (Rahmati-Najarkolaei et al., 2015). Even when individuals believe health behavior reduces disease risk, barriers such as costs and inconvenience may deter them from taking action. Greater perceived barriers may reduce the likelihood of action. The concept of self-efficacy (Bandura, 1997), defined as the confidence or expectation in one’s ability to perform the necessary actions to achieve desired outcomes, was added to the original model as a separate construct (Rosenstock et al., 1988). Self-efficacy predicts healthy behavior, and individuals who are confident in their ability to perform healthy behavior are more likely to engage in it than those without such confidence (Bandura, 1997).

### 2.2. Perceived Susceptibility and PA Participation Intention

Perceived susceptibility is defined as an individual’s belief about the likelihood of being exposed to a specific health problem (Janz & Becker, 1984) and has been discussed as a correlate of intention to participate in PA. Prior studies have suggested that higher perceived susceptibility may be associated with stronger exercise-related intention in some populations. For example, Khodaveisi et al. (2021) found that education based on the HBM improved physical activity-related outcomes, supporting the relevance of susceptibility-related beliefs in exercise contexts. Some earlier work has also suggested that older adults who recognize greater chronic disease risk may be more likely to intend to engage in physical activity (Gristwood, 2011). Taken together, prior evidence suggests that susceptibility beliefs may be related to PA intention, but findings vary by population and health context; therefore, we examine this association in South Korean older adults.

**Hypothesis** **1.** *Higher perceived susceptibility will be positively associated with PA participation intention among older adults.*

### 2.3. Perceived Severity and PA Participation Intention

Perceived severity is defined as an individual’s awareness of the seriousness of a health problem’s consequences (Janz & Becker, 1984). Rahmati-Najarkolaei and colleagues (2015) reported that perceived severity was significantly associated with PA-related outcomes in their application of HBM constructs. More recently, Zhang et al. (2024) found that physical health beliefs were positively associated with college students’ exercise intention, and that this association was partly explained by exercise imagery, suggesting that health-risk–related cognitions can be linked to exercise motivation. Intervention evidence also supports the relevance of health-belief–based approaches, as Xu et al. (2025) reported that an HBM-informed e-education program improved exercise management outcomes in women with gestational hypertension. Overall, evidence suggests severity-related beliefs may be linked to PA intention in some contexts, but effects appear population- and context-dependent; thus, we test this association among older adults in South Korea.

**Hypothesis** **2.** *Higher perceived severity will be positively associated with PA participation intention among older adults.*

### 2.4. Perceived Benefits and PA Participation Intention

Perceived benefit refers to the belief in positive results that can be obtained through PA and has been proven to be one of the strongest predictors of the intention to participate in PA (Janz & Becker, 1984). In older adulthood, this construct may be especially relevant because physical activity can support independence, physical functioning, and healthy aging. Recent reviews indicate that older adults perceive both health-related and psychosocial benefits from physical activity, although the relative importance of these perceived benefits may vary across settings and individuals (Kilgour et al., 2024; Meredith et al., 2023). At the same time, recognizing benefits may not be sufficient to generate intention when barriers remain high or confidence is low, suggesting that the influence of perceived benefits may depend on how it operates alongside other HBM constructs, especially barriers and self-efficacy (Janz & Becker, 1984; Kilgour et al., 2024). Accordingly, although perceived benefits are theoretically relevant to physical activity intention, their empirical association may depend on how they operate alongside other HBM constructs, especially barriers and self-efficacy.

**Hypothesis** **3.** *Higher perceived benefits will be positively associated with PA participation intention among older adults.*

### 2.5. Perceived Barriers and PA Participation Intention

Perceived barriers refer to the recognition of tangible and intangible negative factors or obstacles that may arise when engaging in specific actions (Janz & Becker, 1984). These commonly identified perceived barriers to physical activity among older adults include physical factors (e.g., chronic pain, physical limitations), motivational factors (e.g., dislike of activity, lack of motivation, lack of interest), environmental factors (e.g., unsafe exercise facilities), and psychological factors (e.g., fear of feeling inadequate, worries about what other people think, fear of injury) (Rai et al., 2020). Numerous studies highlight the significant role of barriers in reducing PA intentions. For example, Rai et al. (2020) identified environmental and psychosocial constraints as important factors influencing physical activity participation among older adults with low activity levels. More recently, a systematic review of adults aged 70 years and older (Kilgour et al., 2024) found that concern about physical health and fitness, lack of motivation or interest, fear of falls, and environmental barriers were among the most frequently reported obstacles to physical activity. These findings suggest that perceived barriers are especially salient in later life and may meaningfully reduce physical activity intention. Given the consistent evidence that barriers are inversely associated with PA intentions and the relevance of feasibility constraints in later life, we test the barrier–intention association in older adults in South Korea.

**Hypothesis** **4.** *Higher perceived barriers will be negatively associated with PA participation intention among older adults.*

### 2.6. Self-Efficacy and PA Participation Intention

Self-efficacy refers to the confidence that an individual can successfully perform a specific behavior and has been identified as the most influential variable in predicting PA participation intention (Bandura, 1997). In PA research, self-efficacy is consistently associated with stronger intention and engagement, particularly among older adults for whom pain, functional limitations, and environmental constraints may challenge participation. Recent evidence supports its relevance in later life: a systematic review and meta-analysis reported a significant positive association between exercise self-efficacy and physical activity among older adults (Xie et al., 2025). In addition, a recent systematic review found that participation in exercise interventions is generally associated with improvements in self-efficacy in adults, suggesting that self-efficacy is a modifiable target for PA promotion (Baghbani et al., 2023). Given that self-efficacy is theoretically and empirically linked to PA-related motivation and behavior, we examine its association with PA participation intention among older adults in South Korea.

**Hypothesis** **5.** *Higher self-efficacy will be positively associated with PA participation intention among older adults.*

### 2.7. PA Participation Intention to PA Participation

PA is defined as any activity that results in energy expenditure above the baseline level due to skeletal muscle contractions (Niyazi et al., 2024; World Health Organization, 2020) and is performed voluntarily, beyond activities essential for daily life (Physical Activity Guidelines Advisory Committee, 2018). In health behavior theories, behavioral intention is commonly regarded as a proximal determinant of actual behavior. Intention reflects the degree to which an individual is willing or plans to perform a specific behavior, and prior research has generally supported a positive association between PA intention and subsequent PA behavior. In particular, meta-analytic evidence based on the Theory of Planned Behavior has shown that intention is significantly associated with health-related behaviors, including physical activity (McEachan et al., 2011). More specifically, a meta-analytic structural equation modeling study reported that PA intention was positively associated with actual PA among adults with physical disabilities (Sur et al., 2022). At the same time, recent scholarship has noted that intention does not always translate fully into behavior, highlighting the well-known intention–behavior gap in physical activity research (Feil et al., 2023; Jekauc et al., 2026). Accordingly, although intention may not completely determine PA participation, it remains one of the most important proximal correlates of behavior. Therefore, in the present study, we examine whether PA participation intention is positively associated with actual PA participation among older Korean adults.

**Hypothesis** **6.** *Higher PA participation intention will be positively associated with actual PA participation among older adults.*

### 2.8. Mediating Role of PA Participation Intention

In this study, the five components of the HBM are posited to have a positive relationship with the intention to participate in PA, which, in turn, has a positive relationship with actual PA participation. Because intention is commonly modeled as a proximal correlate of behavior, we examine whether intention serves as a statistical pathway linking HBM beliefs to PA participation in the hypothesized model. Therefore, we propose the following hypothesis:

**Hypothesis** **7.** *PA participation intention will significantly mediate the associations between the five components of HBM—(a) perceived susceptibility, (b) perceived severity, (c) perceived benefits, (d) perceived barriers, and (e) self-efficacy—and actual PA participation.*

## 3. Materials and Methods

### 3.1. Participants

This study included 408 older adults in South Korea. Among the participants, 58.6% were male, and 48.3% held a bachelor’s degree. The average age was 68.84 years (SD = 4.109, range: 65–92 years). Most participants (80.6%) were married, and 55.6% perceived their economic situation as moderate. Regarding health perceptions, 38.0% and 37.0% perceived their physical health as healthy and moderate, respectively, while 54.2% perceived their mental health as healthy.

### 3.2. Measures

#### 3.2.1. Health Belief Model

Health beliefs regarding PA were measured using an adapted version of the Champion Health Belief Model Scale (Champion, 1984) revised by Jacobs (2002). Items were systematically reworded from colorectal cancer screening to PA while preserving the original construct intent; for example, screening-specific wording was replaced with PA-relevant referents (e.g., “engaging in regular physical activity” or “being physically inactive”). Sample items included: perceived susceptibility (“I am very likely to have health problems due to lack of physical activity”), perceived severity (“The thought that a lack of physical activity may cause health problems threatens me”), perceived benefits (“I think regular physical activity will be effective in maintaining health”), perceived barriers (“I think it would be unpleasant to do physical activity”), and self-efficacy (“I know how to do physical activities regularly”).

To ensure linguistic and cultural appropriateness, the items were translated into Korean and back-translated by independent bilingual translators, reviewed by an expert panel for content and clarity, and pilot-tested with older adults to refine wording. The health motivation subscale was excluded because it reflects a general health orientation and may overlap with proximal motivational constructs (e.g., intention) modeled in this study; therefore, we retained five belief domains most directly relevant to PA decision-making: susceptibility (5 items), severity (7 items), benefits (6 items), barriers (6 items), and self-efficacy (5 items). All items used a 5-point Likert scale (1 = strongly disagree to 5 = strongly agree); higher scores indicate stronger endorsement. Internal consistency (Cronbach’s α) was 0.94 (susceptibility), 0.88 (severity), 0.84 (benefits), 0.86 (barriers), and 0.78 (self-efficacy).

#### 3.2.2. PA Participation Intention

The intention to participate in PA was evaluated using a Korean version of a self-report questionnaire (Hagger et al., 2016; B. Kim & Cheon, 2019), which assessed participants’ likelihood of engaging in PA in the future. Responses were rated on a 5-point Likert scale, capturing participants’ agreement or disagreement with statements regarding their intention to participate in PA activities. The questions on the intention to participate in PA comprise four questions regarding: “effort to participate in regular PA,” “willingness to participate in PA under any circumstances,” and “effort and willingness to continue participating in PA in the future.” Internal consistency for this scale was 0.87 in a previous study (Y. H. Lee et al., 2024) and 0.88 in the current study.

#### 3.2.3. PA

PA was assessed using the International Physical Activity Questionnaire–Short Form (IPAQ; P. H. Lee et al., 2011), which examines activity over the past 7 days. There are two questionnaire types: the long and short forms. For data collection convenience, this study used a self-administered short form. The short form of the IPAQ comprises four areas: leisure time, home or outdoor activities, work-related activities, and transport-related activities, thereby encompassing a broad spectrum of PA. Participants reported the frequency (days per week) and duration (minutes per day) of activities such as walking, moderate-intensity activities, and vigorous-intensity activities. Data from the IPAQ-S were converted to PA levels in MET-minutes using the IPAQ scoring method. The calculation formulas for each activity intensity were as follows (measured in MET, minute/week): walking = 3.3 × time spent walking × days walked; moderate-intensity = 4.0 × time spent on moderate-intensity activities × days of moderate activity; vigorous-intensity = 8.0 × time spent on vigorous-intensity activities × days of vigorous activity; total PA = walking + moderate-intensity + vigorous-intensity.

### 3.3. Covariate

To account for potential confounding in older-adult physical activity research, we included the following covariates: age (years), sex (0 = female, 1 = male), education level (coded ordinally from lower to higher attainment), marital status (0 = not married, 1 = married), economic status (self-reported; coded ordinally from lower to higher), and self-rated physical health (1 = poor to 5 = excellent). These covariates were included as exogenous variables in the SEM to statistically adjust for their potential associations with the focal variables.

### 3.4. Procedure

Participants were recruited through Gallup Korea using an online panel. Gallup invited panel members who met the study eligibility criteria (age ≥ 65 years and residence in South Korea) to participate in an online survey; individuals who did not meet the age criterion (i.e., under 65) were excluded. Recruitment was conducted through voluntary participation within the panel (i.e., a non-probability online panel sample). The research team developed the study protocol and questionnaire, including item selection and wording for all measures, and refined the survey for readability and appropriateness for older adults. The survey was administered anonymously via Gallup’s secure platform. Prior to analysis, the research team screened the dataset for eligibility and completeness and reviewed responses for data quality (e.g., missingness and inconsistent response patterns). Gallup provided a de-identified dataset to the authors for analysis.

### 3.5. Analytic Procedure

The data for this study were analyzed using SPSS 28.0 and AMOS 26.0 as follows. First, frequency and descriptive statistics were examined to characterize participants’ demographics and outcome variables. Second, Pearson’s correlation analysis was conducted to summarize bivariate associations among variables and to screen for unusually high intercorrelations. Multicollinearity was evaluated using variance inflation factors (VIF) and tolerance statistics in SPSS, while reliability analysis was performed to calculate Cronbach’s α coefficients. Third, confirmatory factor analysis (CFA) was conducted to evaluate the measurement model, including convergent and discriminant validity. Model fit was assessed using multiple indices: χ^2^/df (CMIN/DF), the comparative fit index (CFI) and Tucker–Lewis index (TLI) (Bentler & Bonett, 1980), and the root mean square error of approximation (RMSEA) (Steiger, 1990). Following commonly used guidelines, an acceptable fit was indicated by CMIN/DF ≤ 3, CFI and TLI ≥ 0.90, and RMSEA ≤ 0.08 (Hu & Bentler, 1999; Kline, 2023). Fourth, convergent validity was assessed using composite reliability (CR) and average variance extracted (AVE), with thresholds of CR ≥ 0.70 and AVE ≥ 0.50 (Fornell & Larcker, 1981). Finally, SEM was conducted to test the study’s hypotheses.

## 4. Results

### 4.1. Descriptive Statistics, Correlation, and Preliminary Data Analysis

Table 1 displays descriptive statistics and bivariate correlations for the primary variables. Key assumptions about the data were also evaluated. The ratio of cases to observed variables was 10:1, deemed optimal for CFA. No outliers were identified, and Tolerance values ranged from 0.48 to 0.65, and VIF values ranged from 1.53 to 2.09, indicating no evidence of problematic multicollinearity. Finally, all variables were considered appropriate for analysis, as skewness values were <|3.00|and kurtosis values were <|10.00|, as recommended by Kline (2023).

### 4.2. Analysis of the Measurement Model and the Structural Model

The simultaneous CFA demonstrated that the measurement model adequately fit the data, χ^2^/df = 1209.52/503 = 2.40, CFI = 0.92, TLI = 0.91, RMSEA = 0.059 (90% confidence interval [CI]: 0.055, 0.063). The factor loadings for each construct were significant, ranging from 0.49 to 0.92, and all composite reliability ratings exceeded 0.70 (Table 2). Furthermore, convergent validity was evaluated using AVE and CR criteria. Although AVE values for some constructs were below the conventional 0.50 threshold, CR values were acceptable and AVE values exceeded the corresponding squared inter-construct correlations, supporting acceptable convergent/discriminant validity with caution (Fornell & Larcker, 1981; Hair et al., 2019). These findings indicate that the measures had satisfactory psychometric properties.

A maximum likelihood structural equation model was used to evaluate the hypotheses presented in the conceptual framework. To address potential confounding age, sex, education, marital status, economic status, and self-rated health indicators were included as covariates and specified as predictors of both participation intention and PA. The covariate-adjusted structural model demonstrated acceptable fit: χ^2^/df = 1703.28/733 = 2.32 (*p* < 0.01); TLI = 0.89; CFI = 0.88; RMSEA = 0.057 (90% CI 0.053, 0.061). Although RMSEA and χ^2^/df indicated acceptable approximate fit, the incremental fit indices were marginal (CFI = 0.88; TLI = 0.89), slightly below the guideline (Hu & Bentler, 1999). Given the complexity of the measurement and structural model, these indices were interpreted as guidelines rather than strict cutoffs, and results are interpreted with appropriate caution (Kline, 2023). To avoid overfitting, we retained the theoretically specified model rather than applying data-driven post hoc modifications (MacCallum et al., 1992).

**Figure 1:**
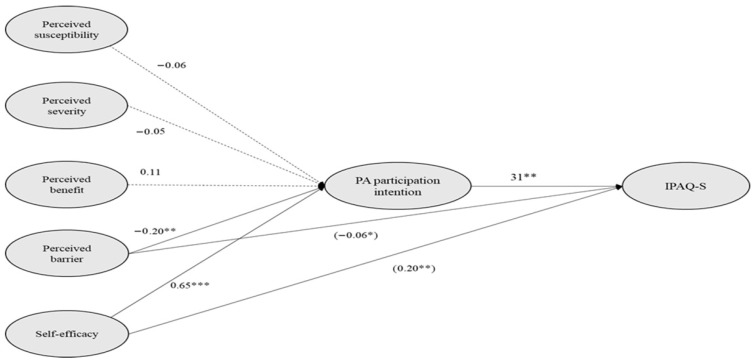
Final structural model. Solid lines indicate significant relationships, whereas the dotted line represents insignificant relationships. Values in parentheses indicate the standardized indirect effects. Significance levels are denoted as follows: *** *p* < 0.001 ** *p* < 0.01, * *p* < 0.05.

The subsequent analysis examined the proposed hypotheses within the conceptual model. As shown in Figure 1, perceived barriers were negatively associated with participation intention (β = −0.21; *p* = 0.002), while self-efficacy was positively associated with participation intention (β = 0.57; *p* < 0.001), consistent with Hypotheses 4 and 5. The path coefficients for perceived susceptibility, perceived severity, and perceived benefit were not significant (*p* > 0.05), providing no support for Hypotheses 1, 2, and 3. Additionally, participation intention was positively associated with the IPAQ-S score (β = 0.31; *p* = 0.02), consistent with Hypothesis 6. Indirect association analyses revealed that participation intention statistically mediated the relationship between perceived barriers and IPAQ-S (β = −0.07; *p* = 0.019) as well as between self-efficacy and IPAQ-S (β = 0.18; *p* < 0.01), partially supporting Hypothesis 7. All covariates were not significant (*ps* > 0.05).

## 5. Discussion

This study aimed to evaluate the predictability of HBM in PA participation among older adults in South Korea. Beyond confirming that barriers and self-efficacy are important, the findings help refine HBM application to PA in later life by suggesting that “actionability” (constraints and perceived capability) may outweigh “risk appraisal” (susceptibility/severity) or generalized benefit beliefs in shaping intention. Specifically, this study’s findings revealed that older adults perceiving fewer barriers to PA participation and greater self-efficacy were more likely to have intentions to participate in PA. Additionally, individuals with higher participation intentions were more likely to report higher levels of actual PA participation. The indirect association pattern further indicates that belief-based factors may be linked to PA through motivational readiness (intention), highlighting intention as a key statistical pathway linking health beliefs to behavior.

First, this study found that perceived barriers were associated with older adults’ PA participation intention, which aligns with several previous studies. Among older adults, commonly reported barriers, such as fear of injury, limited access to age-friendly facilities or programs, financial/transportation constraints, and time or routine-related limitations, may make PA feel impractical or unsafe, thereby weakening intention formation (Kilgour et al., 2024; Meredith et al., 2023; Rai et al., 2020). In later life, such barriers may make PA feel impractical, unsafe, or overly effortful, thereby weakening intention formation. In this sample, these barriers may also capture “everyday feasibility” issues shaped by local environments (e.g., limited age-friendly programs and mobility/transport constraints), suggesting that interventions may be most effective when they reduce friction through safe options, accessible settings, and practical guidance rather than relying primarily on education alone. Theoretically, this pattern supports perceived barriers as a potential “gatekeeper” belief domain for PA in later life: when barriers are salient, intention may remain low even when benefits are acknowledged. Kilgour et al. (2024) identified health concerns, low motivation, fear of falling, and environmental barriers as among the most commonly reported obstacles to physical activity in adults aged 70 and older, while Meredith et al. (Meredith et al., 2023) similarly emphasized the interacting roles of social influences, environmental barriers, and physical limitations in shaping older adults’ engagement in PA.

Second, this study found that self-efficacy was a significant positive associate of PA participation intention among older adults, consistent with extensive literature emphasizing self-efficacy’s key role in health-related behavior (Bandura, 1997). Self-efficacy may be particularly influential for older adults as it may enable them to overcome perceived limitations and external challenges related to PA. Recent evidence reinforces this interpretation. A 2025 systematic review and meta-analysis reported a significant positive association between exercise self-efficacy and physical activity among elderly individuals (Xie et al., 2025), and a 2023 systematic review and meta-analysis found that exercise interventions were generally associated with improvements in self-efficacy and related psychological outcomes in adults (Baghbani et al., 2023). An important contribution of the present study is that, in a context where threat-based beliefs were not significantly associated with intention, self-efficacy emerged as the most actionable cognitive factor for strengthening PA participation intention. This finding suggests that, among older adults, beliefs about one’s capability to engage in PA may be more proximal to intention formation than more distal threat-based beliefs. From a theoretical perspective, these findings reinforce the role of self-efficacy as a central component of HBM-based explanations of PA intention, particularly in older adulthood. Overall, the present results add to the growing literature indicating that self-efficacy is a particularly important cognitive correlate of PA intention in later life.

Interestingly, this study found that perceived benefits did not significantly associate with older adults’ intention to participate in PA, a result that contrasts with much of the existing literature, which identifies perceived benefits as a key motivator for PA intention. One possible explanation is that older adults may already recognize the advantages of PA, such as improved health and quality of life, leading perceived benefits to reach a saturation point where they no longer serve as a distinguishing factor in intention-related differences. Recent reviews indicate that older adults do perceive both health-related and psychosocial benefits from PA, but these perceived advantages coexist with substantial practical, motivational, and contextual constraints (Kilgour et al., 2024; Meredith et al., 2023). In other words, benefits may be cognitively endorsed but behaviorally weak when immediate barriers such as pain, fatigue, weather, safety concerns, or limited access dominate day-to-day decision-making. Thus, in the present context, perceived benefits may operate as a necessary but insufficient belief for intention formation. This null finding should also be interpreted cautiously because the HBM measure was adapted from a colorectal cancer screening instrument. Although the items were reworded and subjected to translation, expert review, and pilot testing, some residual measurement mismatch may have attenuated the observed construct–intention association. Overall, the result suggests that intervention content may need to shift from repeatedly emphasizing long-term benefits toward more concrete barrier-reduction and confidence-building strategies that help translate favorable beliefs into intention and action.

Finally, this study found that perceived threat, encompassing perceived susceptibility and severity, did not significantly associate with older adults’ intentions to participate in PA. Although previous studies report mixed findings regarding the influence of perceived threat on PA intention, the present result suggests that risk appraisal alone may not be sufficient to motivate exercise intention in later life (Janz & Becker, 1984; Khodaveisi et al., 2021; Rahmati-Najarkolaei et al., 2015). One plausible explanation is that some older adults may not readily interpret health risks as cues for behavioral change, particularly when they perceive inactivity-related problems as difficult to modify or not requiring immediate change; population-based evidence has shown that many older adults report that no health behavior change is needed, despite relatively low activity levels (Newsom et al., 2004). Another possibility is that even when older adults acknowledge the risks of inactivity, these threat perceptions may not readily translate into intention if PA still feels difficult, unsafe, or unrealistic. This interpretation is also consistent with fear-appeal research suggesting that threat information is more likely to promote adaptive action when efficacy beliefs are high, whereas low efficacy may instead encourage defensive or avoidant responses (Witte & Allen, 2000). Accordingly, the present findings imply that threat-based beliefs alone may have limited utility for explaining PA intention among older adults unless they are considered alongside more proximal and actionable beliefs, such as perceived barriers and self-efficacy (Kilgour et al., 2024; Xie et al., 2025). As with perceived benefits, these null associations may also partly reflect the challenge of adapting a screening-oriented HBM instrument to the PA context.

This study can offer several important practical implications for PA promotion among older adults, with recommendations anchored to the constructs that showed significant associations in the present model. Because perceived susceptibility, perceived severity, and perceived benefits were not significantly associated with PA participation intention in the present model, the implications below focus on the two belief domains that showed robust associations—perceived barriers and self-efficacy—and on intention as the proximal mechanism linking these beliefs to physical activity. The finding of the negative association between perceived barriers and PA participation intention underscores the need to develop strategies and interventions that mitigate specific barriers to PA among older adults. Common barriers to physical activity participation include health restrictions, fear of strangers, adverse weather, lack of motivation, and a lack of access to facilities (Kilgour et al., 2024; Meredith et al., 2023). Enhancing accessibility to PA facilities, providing subsidies, offering transportation support, and developing PA programs that accommodate individual physical ability variations can reduce limitations preventing older adults from engaging in PA. Healthcare providers can apply these findings to develop targeted interventions or counseling approaches to promote PA among this population. First, providers should first identify and address barriers to PA, such as safety concerns, physical limitations, and unfamiliarity with appropriate activities. They can mitigate these barriers by raising them during consultations and offering practical solutions, including advice on low-intensity PA, referrals to community fitness programs, or physiotherapy sessions.

Additionally, because PA participation intention was positively associated with self-efficacy, the importance of interventions to improve older adults’ confidence in engaging in physical activities should not be overlooked. Techniques for enhancing self-efficacy include allowing individuals to set their own personal goals to increase their confidence and skill in engaging in the physical activity, providing opportunities to observe others participate in the physical activity, reinforcing and providing positive feedback on participants’ performance, and offering an educational program or skill development course to reduce participants’ psychological discomfort associated with the physical activity (Bandura, 1997). These approaches are especially relevant in later life because they can help older adults reframe PA as safe, manageable, and compatible with their current health status. In this sense, messages and programs may be more persuasive when they communicate, explicitly or implicitly, “you can do this safely and gradually,” rather than focusing primarily on generalized health warnings.

Despite its significant theoretical and practical implications, this study has several limitations. First, it employed a cross-sectional research design, which restricts the ability to infer causal relationships among the constructs. In addition, although we tested indirect associations consistent with the hypothesized model, these “statistical pathways” should not be interpreted as causal mediation because temporal ordering cannot be established with cross-sectional data. Future research should consider longitudinal or experimental designs to better explore the dynamic cause–effect relationships among the proposed variables. Second, the self-reported nature of the data may introduce social desirability or recall bias. Older adults may face challenges in accurately recalling their PA participation over the past 7 days. Future studies could utilize objective measures of PA, such as accelerometers or fitness trackers, to enhance the accuracy and robustness of the findings. Third, although the covariate-adjusted SEM showed acceptable approximate fit (e.g., RMSEA and χ^2^/df), the incremental fit indices (CFI/TLI) were marginally below common guidelines; therefore, the structural associations should be interpreted with appropriate caution (Hu & Bentler, 1999; Kline, 2023).

Fourth, the sampling process may limit the generalizability of the findings, as this study focused exclusively on older adults in South Korea. These results may not fully apply to other cultural or socioeconomic contexts. In addition, because participants were recruited through a voluntary online panel, older adults with limited internet access or lower digital literacy may have been underrepresented, which may have introduced selection bias. The sample was also relatively male-dominated and highly educated, which should be considered when interpreting generalizability. Future research should test this conceptual model with diverse populations to explore potential cultural or contextual differences in the relationships between variables.

Fifth, while this study used HBM to identify factors influencing PA participation intention among older adults, other health behavior constructs, such as social support, outcome expectations, subjective norms, and intrinsic motivation, might also play significant roles in predicting PA participation. Future studies should consider integrating other theoretical frameworks, such as social cognitive theory (Bandura, 1997) or self-determination theory (Ryan & Deci, 2000), to provide a more comprehensive understanding of PA participation among older adults.

Finally, this study did not account for the interaction effects among HBM constructs, such as whether perceived benefits could counteract the influence of perceived barriers. Future research should examine these interactions to identify more nuanced intervention strategies tailored to specific subsets of older adults, thereby supporting long-term PA engagement and preserving high levels of activity.

## 6. Conclusions

Physical inactivity and its associated negative outcomes are prevalent among older adults in South Korea, a nation experiencing rapid aging. This study aimed to explore the applicability of the HBM to PA participation intention and PA participation among older adults in South Korea. The findings emphasized the importance of perceived barriers and self-efficacy, as PA participation intention was negatively associated with perceived barriers and positively associated with self-efficacy. However, no significant associations were observed for perceived susceptibility, perceived severity, or perceived benefits. These results advance HBM theory by illustrating the differential associations of its constructs in the context of older adults and PA. Ultimately, the study underscores the necessity of tailored interventions to promote active aging, particularly those aimed at overcoming barriers and enhancing self-efficacy, to support sustained PA participation among older adults.

## Figures and Tables

**Table 1:** Descriptive statistics and correlations among study constructs.

Construct	1	2	3	4	5	6	7
1. Perceived susceptibility	(0.94)						
2. Perceived severity	0.69 **	(0.88)					
3. Perceived benefit	−0.09	0.03	(0.84)				
4. Perceived barrier	0.44 **	0.40 **	−0.38 **	(0.86)			
5. Self-efficacy	−0.22 **	−0.08	0.67 **	−0.34 **	(0.78)		
6. PA participation intention	−0.24 **	−0.10 *	0.54 **	−0.37 **	0.59 **	(0.90)	
7. IPAQ-S	−0.35 **	−0.17 **	0.17 **	−0.07	0.23 **	0.26 **	
*M*	2.93	3.03	4.10	2.21	3.89	4.10	3400.61
*SD*	0.93	0.78	0.48	0.69	0.50	0.60	3166.15
Skewness	0.02	−0.34	−0.89	0.80	−0.91	−0.91	1.89
Kurtosis	−0.71	0.01	2.94	0.66	2.01	3.01	4.56

Notes. *N* = 408; * *p* < 0.05, ** *p* < 0.01. PA, physical activity; IPAQ-S, International Physical Activity Questionnaire–Short Form.

**Table 2:** Psychometric properties of the proposed constructs.

Variable	Item	β	ρ_c_	AVE
Perceived susceptibility	I am very likely to have health problems due to lack of physical activity.	0.86	0.95	0.78
	I think lack of physical activity can cause health problems.	0.86		
	I have the potential to have health problems due to lack of physical activity within 10 years.	0.87		
	I think there are many risk factors for causing health problems due to lack of physical activity.	0.92		
	I think there is a higher possibility of health problems due to lack of physical activity compared to other people.	0.89		
Perceived severity	The thought that a lack of physical activity may cause health problems threatens me.	0.86	0.86	0.47
	When you think of health problems due to lack of physical activity, your pulse rate gets faster.	0.77		
	The thought that a lack of physical activity will cause health problems scares me.	0.86		
	If you have health problems due to lack of physical activity, it will be a problem for a long time.	0.68		
	Health problems caused by lack of physical activity will adversely affect family and interpersonal relationships.	0.49		
	If I have health problems due to lack of physical activity, my whole life is likely to change.	0.50		
	If health problems due to lack of physical activity develop and progress, I think I may die within 5 years.	0.52		
Perceived benefit	I think regular physical activity will be effective in maintaining health.	0.68	0.84	0.47
	I get to worry less about my health by doing physical activities.	0.65		
	I think regular physical activity is effective in preventing health problems.	0.74		
	I think physical activity reduces the chances of dying from chronic diseases.	0.70		
	If I do physical activities regularly, I think that serious treatment or surgery can be reduced, even if health problems arise.	0.62		
	I think physical activity helps prevent health problems.	0.73		
Perceived barrier	I feel uncomfortable talking about lack of physical activity.	0.61	0.86	0.51
	If I do physical activities regularly, I think I will be more worried about my health.	0.71		
	I think regular physical activity might make me feel uncomfortable	0.78		
	It takes too much time to do regular physical activity.	0.66		
	I think it would be unpleasant to do physical activity.	0.81		
	Physical activity would cost a lot of money	0.69		
Self-efficacy	I know how to do physical activities regularly.	0.61	0.78	0.42
	If necessary, I will do regular physical activity.	0.77		
	If I had a health problem, I would do physical activity more steadily.	0.67		
	I can know the normal/abnormal changes in my physical strength or physical condition.	0.54		
	If I do physical activity regularly, I think I can prevent health problems early.	0.63		
PA participation intention	I will participate in physical activities in the future	0.83	0.87	0.63
	I have plans to reengage in physical activity	0.72		
	I will continue to participate in physical activities	0.83		
	I have an intention to participate in physical activities in the future	0.80		

## Data Availability

The datasets generated and analyzed during the current study are available from the corresponding author on reasonable request.
